# Editorial: Therapeutic management of COVID-19 in immunocompromised patients and interaction with the immune response: from preventive to curative strategies

**DOI:** 10.3389/fimmu.2024.1441199

**Published:** 2024-07-16

**Authors:** Ilies Benotmane, Morgane Solis

**Affiliations:** ^1^ Département de Néphrologie-Dialyse-Transplantation, Hôpitaux Universitaires de Strasbourg, Strasbourg, France; ^2^ Laboratoire de Virologie, Hôpitaux Universitaires de Strasbourg, Strasbourg, France

**Keywords:** COVID-19, SARS-CoV-2, immunocompromised patients, solid organ transplantation, HIV, vaccine, monoclonal antibody

Immunocompromised patients suffer from a higher rate of COVID-19-related complications and poor outcomes, including hospitalization, intensive care unit admission, and mortality (1). Despite the administration of billions of vaccine doses, these patients remain at significant risk due to poor immune responses (2), making COVID-19 an ongoing concern. Monoclonal antibodies have shown preventative and curative benefits (3, 4); however, the high mutation rate of SARS-CoV-2, leading to numerous escape variants, has rendered most of these treatments ineffective (5). Moreover, the use of nirmatrelvir/ritonavir and remdesivir in patients with chronic renal failure—a common comorbidity in immunocompromised patients—is challenging, and can interfere with the pharmacokinetics of immunosuppressive drugs (6). Thus, the management of COVID-19 in immunocompromised patients remains complex.

This Research Topic comprises eight articles that explore various aspects of COVID-19 therapeutic management and impact on the immune response in immunocompromised patients.

The COVID-19 pandemic accelerated the development of vaccines, including novel platforms like mRNA vaccines. Despite these advancements, data have shown a weak vaccine response in immunocompromised individuals. Hoek et al. conducted a 6-month follow-up study after the second vaccination, examining the humoral and cellular immune responses of lung transplant recipients and patients on the waiting list, compared to controls. Antibody levels and T-cell responses were lower in lung transplant recipients compared to patients on the waiting list, and both groups had lower levels than the controls. Six months post-second vaccination, all groups exhibited a decline in antibody titers and T-cell responses. These findings underscore the importance of ensuring protection for patients awaiting lung transplants.

Various strategies have been investigated to enhance vaccine response in immunocompromised patients. In a meta-analysis, Wang et al. compared the effectiveness of the mRNA-1273 and BNT162b2 COVID-19 vaccines in immunocompromised individuals. Their data included 17 studies with a population of 178,298 and 170,760 patients receiving mRNA-1273 and BNT162b2, respectively. The mRNA-1273 vaccine was associated with improved clinical effectiveness in immunocompromised populations compared to BNT162b2, significantly reducing the risk of SARS-CoV-2 infection, severe infection, COVID-19-associated hospitalization, and mortality. Interestingly, Hung et al. studied the efficacy of an additional vaccine dose in people living with HIV (PLWH) with mild immunodeficiency compared to healthy non-HIV individuals. The third shot significantly boosted SARS-CoV-2 immunity, with better antibody responses and higher IFN-γ and IL-2 responses of the CD4+ and CD8+ T cells in PLWH compared to those without HIV. These results suggest that slightly compromised immunity in PLWH retains the functional capacity for an enhanced response to a third vaccine dose or natural infection.

Subsequently, Yendewa et al. discussed the unclear risk of post-acute sequelae of SARS-CoV-2 (PASC) in immunocompromised patients. HIV-positive status increased the odds of PASC, while COVID-19 vaccination reduced the risks of PASC and all-cause mortality in people with HIV (PWH).

Several contributions focused on monoclonal antibodies, their protective potential, and immune pressure on viral fitness and neutralization escape. Pulvirenti et al. described tixagevimab/cilgavimab prophylaxis in patients with inborn errors of immunity. The effectiveness of this combination during the Omicron wave was 85% against infection and 82% against symptomatic disease within 90 days post-administration but became null thereafter. The protection provided by these mAbs was significantly inferior compared to recently infected patients and was lost with newer viral variants. Viriyakitkosol et al. showed that BA.2 and BA.5 variants could evade neutralization by human monoclonal antibodies. Continuous *in vitro* exposure to the antibody induced the A475V mutation in the spike protein, substantially reducing the neutralization activity for both monoclonal antibodies and human sera, highlighting the evolutionary process of SARS-CoV-2 under selection pressure. As a counterpoint, Abreu et al. presented custom-designed multibodies that could neutralize SARS-CoV-2 in a variant-insensitive manner. This pentameric scaffold, based on a mammalian protein, could be customized with several protein-binding modules to neutralize SARS-CoV-2 spike proteins from one or multiple viral particles. *In vitro*, this construct’s neutralization potential surpassed the activity of sera from vaccinated patients, though *in vivo* protection has yet to be assessed.

Finally, Furuya et al. described the curative treatment of persistent COVID-19 by ensitrelvir in two lymphoma patients. These patients underwent successive rounds of treatment with molnupiravir, remdesivir, and monoclonal antibodies. Administration of ensitrelvir improved clinical status and viral load became undetectable within days. This novel drug inhibits SARS-CoV-2 3-chymotrypsin-like protease, essential for replication, and represents a promising treatment approach.

As outlined in the editorial, the present Research Topic includes many aspects of COVID-19 therapeutic management and immune responses in immunocompromised patients, encompassing vaccine responses and strategies, monoclonal antibody usage, viral evasion risks, and new antiviral drugs. These findings contribute to the current knowledge of preventive and curative strategies in immunocompromised patients and will aid in improving the therapeutic management of COVID-19.

## Author contributions

IB: Writing – original draft, Writing – review & editing. MS: Writing – original draft, Writing – review & editing.
